# (*E*)-2,4,6-Trimethyl-*N*-(pyridin-2-yl­methyl­idene)aniline

**DOI:** 10.1107/S1600536812015905

**Published:** 2012-04-18

**Authors:** Yu-Wei Dong, Rui-Qing Fan, Ping Wang, Yu-Lin Yang

**Affiliations:** a Department of Chemistry, Harbin Institute of Technology, Harbin 150001, People’s Republic of China

## Abstract

In the title compound, C_15_H_16_N_2_, has an *E* conformation about the central N=C bond. The benzene and pyridine rings are almost normal to one another with a dihedral angle of 87.47(8)°. In the crystal, there are no classical hydrogen bonds.

## Related literature

For C—N bond forming reactions, see: Alonso-Moreno *et al.* (2009 ▶); Qiu *et al.* (2009 ▶). For imino C=N bonds in a related structure, see: Nienkemper *et al.* (2006 ▶). For the preparation of related compounds, see: Bianchini *et al.* (2001 ▶); Fan *et al.* (2009 ▶).
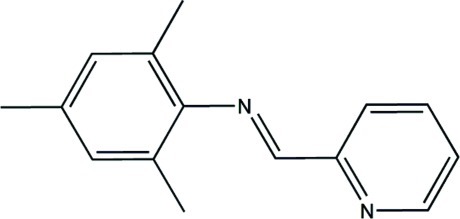


## Experimental

### 

#### Crystal data

C_15_H_16_N_2_
*M* *_r_* = 224.30
Monoclinic, 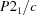

*a* = 8.2490 (16) Å
*b* = 16.136 (3) Å
*c* = 10.150 (2) Å
β = 104.76 (3)°
*V* = 1306.4 (4) Å^3^
*Z* = 4
Mo *K*α radiation
μ = 0.07 mm^−1^
*T* = 293 K
0.36 × 0.34 × 0.29 mm

#### Data collection

Bruker SMART APEX CCD area-detector diffractometer
Absorption correction: multi-scan (*SADABS*; Bruker, 2000 ▶) *T*_min_ = 0.976, *T*_max_ = 0.981
12591 measured reflections
2982 independent reflections
1952 reflections with *I* > 2σ(*I*)
*R*_int_ = 0.044

#### Refinement

*R*[*F* ^2^ > 2σ(*F* ^2^)] = 0.056
*wR*(*F* ^2^) = 0.200
*S* = 1.03
2982 reflections
154 parameters
H-atom parameters constrained
Δρ_max_ = 0.22 e Å^−3^
Δρ_min_ = −0.22 e Å^−3^

### 

Data collection: *SMART* (Bruker, 2000 ▶); cell refinement: *SAINT* (Bruker, 2000 ▶); data reduction: *SAINT*; program(s) used to solve structure: *SHELXS97* (Sheldrick, 2008 ▶); program(s) used to refine structure: *SHELXL97* (Sheldrick, 2008 ▶); molecular graphics: *SHELXP97* (Sheldrick, 2008 ▶); software used to prepare material for publication: *SHELXTL* (Sheldrick, 2008 ▶).

## Supplementary Material

Crystal structure: contains datablock(s) global, I. DOI: 10.1107/S1600536812015905/zj2067sup1.cif

Structure factors: contains datablock(s) I. DOI: 10.1107/S1600536812015905/zj2067Isup2.hkl

Supplementary material file. DOI: 10.1107/S1600536812015905/zj2067Isup3.cml

Additional supplementary materials:  crystallographic information; 3D view; checkCIF report

## Figures and Tables

**Fig. 1.**
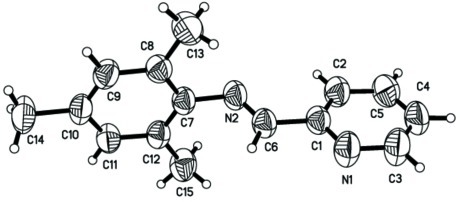
View of the molecule of (I) showing the atom-labelling scheme. Displacement ellipsoids are drawn at the 50% probability level.

**Fig. 2.**
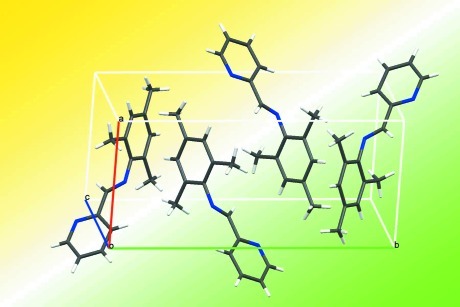
Packing of (I) along c axis direction.

## supplementary crystallographic information

### Comment

C—N bond forming reactions are of considerable interest in both synthetic organic due to the importance of amines and their derivatives in almost all areas of chemistry (Alonso-Moreno *et al.*, 2009, Qiu *et al.*, 2009). It is still challenging to design and rationally synthesize ligand with unique structures and functions. For this regard, we reported the crystal structure of compound (I). The molecular structure of (I) is shown in Fig. 1 and selected bond distances are given in Table 1. The imino C==N bonds have typical double-bond characteristic with bond lengths of 1.240 (2), which are similar to that in (2,6-diisopropylphenyl)[1-(pyridin-2-yl)ethylidene]amine, 1.280 (2) Å (Nienkemper *et al.*, 2006). The compound (I) possesses a structure with approximate *P*2_1_/*c* symmetry. The dihedral angles between 2,4,6- trimethyl-substituted phenyl rings and the pyridine ring are 87.5° respectively.

### Experimental

The Schiff base was prepared according to the literature methods for analogous compounds (Fan *et al.*, 2009, Bianchini *et al.*, 2002). Pyridine-2-carboxaldehyde (1.69 g, 15.8 mmol) and 2,4,6-trimethyaniline (2.13 g, 15.7 mmol) were dissolved in 20 ml of methanol containing a few drops of formic acid and the resulting mixture was heated at reflux temperature for 4 h. Partial evaporation of solvent under reduced pressure gave yellow soild.Yellow block crystals suitable for X-ray diffraction analysis were obtained by recrystallization from n-hexane,and the specific method was that a solution of yellow soild in 15 ml of n-hexane was heated at 338 K and then allowed to cool down to room temperature.Yield:76% (2.68 g).

### Refinement

The C-bound H atoms were positioned geometrically with C—H = 0.93–0.96 Å, and allowed to ride on their parent atoms with *U*_iso_(H) = 1.2 *U*_eq_(C) for CH_2_ groups, and 1.5 *U*_eq_(C) for CH_3_ groups.

### Crystal data

**Table:**

C_15_H_16_N_2_	*F*(000) = 480
*M**_r_* = 224.30	*D*_x_ = 1.140 Mg m^−^^3^
Monoclinic, *P*2_1_/*c*	Mo *K*α radiation, λ = 0.71073 Å
Hall symbol: -P 2ybc	Cell parameters from 12591 reflections
*a* = 8.2490 (16) Å	θ = 3.1–27.5°
*b* = 16.136 (3) Å	µ = 0.07 mm^−^^1^
*c* = 10.150 (2) Å	*T* = 293 K
β = 104.76 (3)°	Block, colorless
*V* = 1306.4 (4) Å^3^	0.36 × 0.34 × 0.29 mm
*Z* = 4	

### Data collection

**Table:**

Bruker SMART APEX CCD area-detector diffractometer	2982 independent reflections
Radiation source: fine-focus sealed tube	1952 reflections with *I* > 2σ(*I*)
Graphite monochromator	*R*_int_ = 0.044
phi and ω scans	θ_max_ = 27.5°, θ_min_ = 3.1°
Absorption correction: multi-scan (*SADABS*; Bruker, 2000)	*h* = −10→10
*T*_min_ = 0.976, *T*_max_ = 0.981	*k* = −20→20
12591 measured reflections	*l* = −13→13

### Refinement

**Table:**

Refinement on *F*^2^	Primary atom site location: structure-invariant direct methods
Least-squares matrix: full	Secondary atom site location: difference Fourier map
*R*[*F*^2^ > 2σ(*F*^2^)] = 0.056	Hydrogen site location: inferred from neighbouring sites
*wR*(*F*^2^) = 0.200	H-atom parameters constrained
*S* = 1.03	*w* = 1/[σ^2^(*F*_o_^2^) + (0.1315*P*)^2^] where *P* = (*F*_o_^2^ + 2*F*_c_^2^)/3
2982 reflections	(Δ/σ)_max_ = 0.017
154 parameters	Δρ_max_ = 0.22 e Å^−^^3^
0 restraints	Δρ_min_ = −0.22 e Å^−^^3^

### Special details

**Table:**

Geometry. All e.s.d.'s (except the e.s.d. in the dihedral angle between two l.s. planes) are estimated using the full covariance matrix. The cell e.s.d.'s are taken into account individually in the estimation of e.s.d.'s in distances, angles and torsion angles; correlations between e.s.d.'s in cell parameters are only used when they are defined by crystal symmetry. An approximate (isotropic) treatment of cell e.s.d.'s is used for estimating e.s.d.'s involving l.s. planes.
Refinement. Refinement of *F*^2^ against ALL reflections. The weighted *R*-factor *wR* and goodness of fit *S* are based on *F*^2^, conventional *R*-factors *R* are based on *F*, with *F* set to zero for negative *F*^2^. The threshold expression of *F*^2^ > σ(*F*^2^) is used only for calculating *R*-factors(gt) *etc*. and is not relevant to the choice of reflections for refinement. *R*-factors based on *F*^2^ are statistically about twice as large as those based on *F*, and *R*- factors based on ALL data will be even larger.

### Fractional atomic coordinates and isotropic or equivalent isotropic displacement parameters (Å^2^)

**Table:**

	*x*	*y*	*z*	*U*_iso_*/*U*_eq_	
N1	−0.08242 (19)	−0.04738 (10)	0.77519 (15)	0.0748 (5)	
N2	0.20612 (16)	0.11712 (9)	0.82639 (12)	0.0612 (4)	
C1	−0.02245 (18)	0.02378 (10)	0.74182 (15)	0.0559 (4)	
C2	−0.0893 (2)	0.06280 (13)	0.61937 (16)	0.0714 (5)	
H2A	−0.0460	0.1131	0.5993	0.086*	
C3	−0.2097 (2)	−0.08143 (14)	0.6828 (2)	0.0830 (6)	
H3A	−0.2515	−0.1318	0.7042	0.100*	
C4	−0.2827 (2)	−0.04703 (15)	0.55889 (18)	0.0816 (6)	
H4A	−0.3718	−0.0731	0.4983	0.098*	
C5	−0.2218 (3)	0.02585 (16)	0.52670 (19)	0.0854 (6)	
H5A	−0.2686	0.0509	0.4431	0.102*	
C6	0.12079 (18)	0.05750 (10)	0.84678 (15)	0.0572 (4)	
H6A	0.1483	0.0328	0.9324	0.069*	
C7	0.34417 (17)	0.14354 (9)	0.93500 (15)	0.0529 (4)	
C8	0.50580 (18)	0.11695 (10)	0.93612 (15)	0.0561 (4)	
C9	0.63854 (18)	0.14503 (10)	1.04089 (16)	0.0598 (4)	
H9A	0.7465	0.1274	1.0430	0.072*	
C10	0.61645 (18)	0.19772 (10)	1.14131 (17)	0.0600 (4)	
C11	0.45476 (19)	0.22368 (11)	1.13626 (16)	0.0611 (4)	
H11A	0.4379	0.2595	1.2033	0.073*	
C12	0.31661 (18)	0.19793 (10)	1.03427 (15)	0.0556 (4)	
C13	0.5355 (2)	0.05931 (13)	0.82917 (18)	0.0760 (5)	
H13A	0.6532	0.0479	0.8462	0.114*	
H13B	0.4964	0.0846	0.7411	0.114*	
H13C	0.4758	0.0085	0.8316	0.114*	
C14	0.7641 (2)	0.22552 (14)	1.2553 (2)	0.0844 (6)	
H14A	0.8654	0.2017	1.2422	0.127*	
H14B	0.7479	0.2076	1.3413	0.127*	
H14C	0.7723	0.2849	1.2546	0.127*	
C15	0.1431 (2)	0.22807 (13)	1.0332 (2)	0.0755 (6)	
H15A	0.1494	0.2642	1.1094	0.113*	
H15B	0.0730	0.1815	1.0393	0.113*	
H15C	0.0965	0.2577	0.9501	0.113*	

### Atomic displacement parameters (Å^2^)

**Table:**

	*U*^11^	*U*^22^	*U*^33^	*U*^12^	*U*^13^	*U*^23^
N1	0.0668 (9)	0.0740 (10)	0.0716 (9)	−0.0142 (7)	−0.0044 (7)	0.0011 (7)
N2	0.0550 (7)	0.0706 (9)	0.0494 (7)	−0.0078 (6)	−0.0024 (6)	0.0029 (6)
C1	0.0461 (7)	0.0634 (9)	0.0540 (8)	0.0000 (6)	0.0050 (6)	−0.0066 (7)
C2	0.0625 (9)	0.0853 (13)	0.0584 (9)	−0.0121 (8)	0.0006 (8)	0.0021 (8)
C3	0.0724 (11)	0.0809 (13)	0.0839 (13)	−0.0218 (9)	−0.0016 (10)	−0.0076 (10)
C4	0.0599 (10)	0.1080 (16)	0.0677 (11)	−0.0180 (10)	−0.0007 (9)	−0.0236 (11)
C5	0.0723 (12)	0.1173 (17)	0.0541 (9)	−0.0139 (11)	−0.0067 (8)	0.0006 (10)
C6	0.0515 (8)	0.0627 (9)	0.0499 (7)	−0.0020 (7)	−0.0008 (6)	0.0013 (7)
C7	0.0489 (7)	0.0565 (9)	0.0470 (7)	−0.0056 (6)	0.0006 (6)	0.0057 (6)
C8	0.0537 (8)	0.0609 (9)	0.0506 (8)	−0.0007 (7)	0.0077 (6)	0.0032 (6)
C9	0.0429 (7)	0.0695 (10)	0.0631 (9)	−0.0015 (6)	0.0062 (7)	0.0033 (7)
C10	0.0473 (8)	0.0654 (10)	0.0600 (9)	−0.0091 (7)	0.0001 (7)	−0.0011 (7)
C11	0.0556 (8)	0.0634 (10)	0.0593 (8)	−0.0052 (7)	0.0055 (7)	−0.0090 (7)
C12	0.0463 (7)	0.0590 (9)	0.0564 (8)	−0.0009 (6)	0.0038 (6)	0.0015 (7)
C13	0.0742 (11)	0.0847 (13)	0.0654 (10)	0.0051 (9)	0.0113 (9)	−0.0099 (9)
C14	0.0577 (10)	0.0992 (15)	0.0829 (12)	−0.0133 (9)	−0.0066 (9)	−0.0186 (11)
C15	0.0522 (9)	0.0831 (12)	0.0836 (12)	0.0113 (8)	0.0035 (8)	−0.0091 (10)

### Geometric parameters (Å, º)

**Table:**

N1—C1	1.328 (2)	C8—C13	1.496 (2)
N1—C3	1.335 (2)	C9—C10	1.375 (2)
N2—C6	1.240 (2)	C9—H9A	0.9300
N2—C7	1.4333 (18)	C10—C11	1.386 (2)
C1—C2	1.377 (2)	C10—C14	1.518 (2)
C1—C6	1.478 (2)	C11—C12	1.393 (2)
C2—C5	1.383 (2)	C11—H11A	0.9300
C2—H2A	0.9300	C12—C15	1.509 (2)
C3—C4	1.366 (3)	C13—H13A	0.9600
C3—H3A	0.9300	C13—H13B	0.9600
C4—C5	1.351 (3)	C13—H13C	0.9600
C4—H4A	0.9300	C14—H14A	0.9600
C5—H5A	0.9300	C14—H14B	0.9600
C6—H6A	0.9300	C14—H14C	0.9600
C7—C12	1.398 (2)	C15—H15A	0.9600
C7—C8	1.398 (2)	C15—H15B	0.9600
C8—C9	1.394 (2)	C15—H15C	0.9600
			
C1—N1—C3	117.01 (15)	C8—C9—H9A	118.7
C6—N2—C7	118.42 (13)	C9—C10—C11	117.87 (14)
N1—C1—C2	122.42 (15)	C9—C10—C14	121.05 (15)
N1—C1—C6	114.57 (13)	C11—C10—C14	121.08 (17)
C2—C1—C6	123.01 (16)	C10—C11—C12	122.30 (16)
C1—C2—C5	118.83 (19)	C10—C11—H11A	118.8
C1—C2—H2A	120.6	C12—C11—H11A	118.8
C5—C2—H2A	120.6	C11—C12—C7	118.05 (14)
N1—C3—C4	124.2 (2)	C11—C12—C15	120.26 (16)
N1—C3—H3A	117.9	C7—C12—C15	121.68 (14)
C4—C3—H3A	117.9	C8—C13—H13A	109.5
C5—C4—C3	118.21 (16)	C8—C13—H13B	109.5
C5—C4—H4A	120.9	H13A—C13—H13B	109.5
C3—C4—H4A	120.9	C8—C13—H13C	109.5
C4—C5—C2	119.27 (17)	H13A—C13—H13C	109.5
C4—C5—H5A	120.4	H13B—C13—H13C	109.5
C2—C5—H5A	120.4	C10—C14—H14A	109.5
N2—C6—C1	123.27 (14)	C10—C14—H14B	109.5
N2—C6—H6A	118.4	H14A—C14—H14B	109.5
C1—C6—H6A	118.4	C10—C14—H14C	109.5
C12—C7—C8	121.12 (13)	H14A—C14—H14C	109.5
C12—C7—N2	119.87 (13)	H14B—C14—H14C	109.5
C8—C7—N2	118.96 (14)	C12—C15—H15A	109.5
C9—C8—C7	117.96 (15)	C12—C15—H15B	109.5
C9—C8—C13	120.92 (14)	H15A—C15—H15B	109.5
C7—C8—C13	121.12 (14)	C12—C15—H15C	109.5
C10—C9—C8	122.69 (14)	H15A—C15—H15C	109.5
C10—C9—H9A	118.7	H15B—C15—H15C	109.5

## References

[bb1] Alonso-Moreno, C., Carrillo-Hermosilla, F., Romero-Fernández, J., Rodríguez, A. M., Otero, A. & Antiñolo, A. (2009). *Adv. Synth. Catal.* **351**, 881–890.

[bb2] Bianchini, C., Lee, H. M., Mantovani, G., Meli, A. & Oberhauser, W. (2001). *New J. Chem.* **26**, 387–397.

[bb3] Bruker (2000). *SMART*, *SAINT* and *SADABS* Bruker AXS Inc., Madison, Wisconsin, USA.

[bb4] Fan, R. Q., Yang, Y. L., Yin, Y. B., Hasi, W. L. J. & Mu, Y. (2009). *Inorg. Chem.* **48**, 6034–6043. 10.1021/ic900339u 19492826

[bb5] Nienkemper, K., Kotov, V. V., Kehr, G., Erker, G. & Fröhlich, R. (2006). *Eur. J. Inorg. Chem.* pp. 366–379.

[bb6] Qiu, C. J., Zhang, Y. C., Gao, Y. & Zhao, J. Q. (2009). *J. Organomet. Chem.* **694**, 3418–3424.

[bb7] Sheldrick, G. M. (2008). *Acta Cryst.* A**64**, 112–122. 10.1107/S0108767307043930 18156677

